# Dosimetric Coverage of the External Anal Sphincter by 3-Dimensional Conformal Fields in Rectal Cancer Patients Receiving Neoadjuvant Chemoradiation: Implications for the Concept of Sphincter-Preserving Radiation Therapy

**DOI:** 10.1155/2014/578243

**Published:** 2014-06-25

**Authors:** Yi-Jen Chen, Michelle B. Chen, Alan J. Liu, Julian Sanchez, Peter Tsai, An Liu

**Affiliations:** ^1^Radiation Oncology, City of Hope Medical Center, 1500 East Duarte Road, Duarte, CA 91010, USA; ^2^Colorectal Surgery, City of Hope Medical Center, 1500 East Duarte Road, Duarte, CA 91010, USA

## Abstract

*Background*. We evaluated the anatomic location of the external anal sphincter (EAS) to pelvic bony landmarks related to 3-dimensional conformal radiotherapy (3DRT) and studied the dosimetric coverage of the EAS in patients undergoing neoadjuvant chemoradiation for rectal cancer. *Methods*. Sixty-four consecutive rectal cancer patients treated with neoadjuvant chemoradiation were included. All patients were treated in a prone position on a bellyboard by 3DRT. The inferior border of the RT fields was at least 3–5 cm inferior to the gross tumorous volume (GTV) or at the inferior border of the obturator foramen (IBOF), whichever was more inferior. The EAS was contoured and dose distributions were determined using dose-volume histograms. *Results*. In 53 out of 64 cases (82.8%), the EAS was completely inferior to the IBOF. In the remaining 11 cases, the EAS was either overlapping the IBOF (10 cases; 15.6%) or completely superior to the IBOF (1 case; 1.7%). The average mean dose delivered to the EAS was 2795 cGy. Lower mean doses were delivered to the EAS when the center of the EAS was located more distant from the GTV. *Conclusions*. Meticulous planning to define the inferior border of the RT field is recommended to avoid irradiating the EAS.

## 1. Introduction

Neoadjuvant chemoradiation (CRT) followed by surgical intervention is recommended for patients with stage II or III rectal cancer. Compared to postoperative CRT, neoadjuvant CRT is associated with a significantly reduced local recurrence, reduced treatment-related acute and chronic toxicity, and an increased rate of sphincter preservation [1]. Although treatment-related toxicity was reduced, neoadjuvant CRT still caused 40% acute and 24% chronic grade 3 or 4 toxicity. Recently, Bruheim et al. compared patients without a stoma who were treated by pre- or postoperative CRT or radiotherapy (RT) to patients who had surgery alone. The study confirmed that patients who had CRT or RT experienced significantly poorer long-term effects on anorectal function, especially in terms of bowel frequency, urgency, and fecal incontinence, which negatively impacted their quality of life [2].

The design and delivery of pelvic RT for patients with rectal cancer are based on the anatomic location of the cancer, the pathways of lymphatic spreading, and patterns of cancer recurrence. Techniques including multiple-field RT and placing the patient in a prone position are generally used to reduce RT toxicity to the small intestine [3]. To define RT fields, margins from primary cancer are included to make sure the targets are well covered. General guidelines indicate that the inferior border of the RT field should be at least 3 to 5 cm inferior to the primary tumor or at the inferior border of the obturator foramen (IBOF), whichever is more inferior [4]. Depending on the location of the primary tumor and its anatomic relation to the external anal sphincter (EAS), the EAS could be located within, at the border of, or outside the RT fields.

Rectal sensation, rectal storage capacity, and sphincter pressure determine normal anorectal continence. The EAS is the muscle that contracts to prevent stool or air from leaving the rectum. Although the oncologic benefit of treating rectal cancer with RT is confirmed, the functional impact on the EAS related to dosimetric coverage of the radiation remains undetermined. We hypothesized that standard three-dimensional RT (3DRT) for patients with rectal cancer would inevitably deliver certain doses of radiation to the EAS, which could contribute to long-term anorectal dysfunction. The purpose of this study was to define the anatomic location of EAS relative to the pelvic bony landmarks by 3DRT. Secondly, dosimetric coverage of the EAS was determined in patients with rectal cancer in varied locations.

## 2. Materials and Methods

The data from 64 consecutive stages I, II, and III rectal cancer patients undergoing neoadjuvant CRT at City of Hope Medical Center were analyzed retrospectively. The study was approved by the Institutional Review Board at City of Hope Medical Center. The CT simulation imaging and RT dosimetric plans of each patient were analyzed to define the anatomic location of EAS related to the IBOF. In addition, the radiation dosimetric coverage of the EAS by 3DRT was determined in each case.

**Figure 1:**
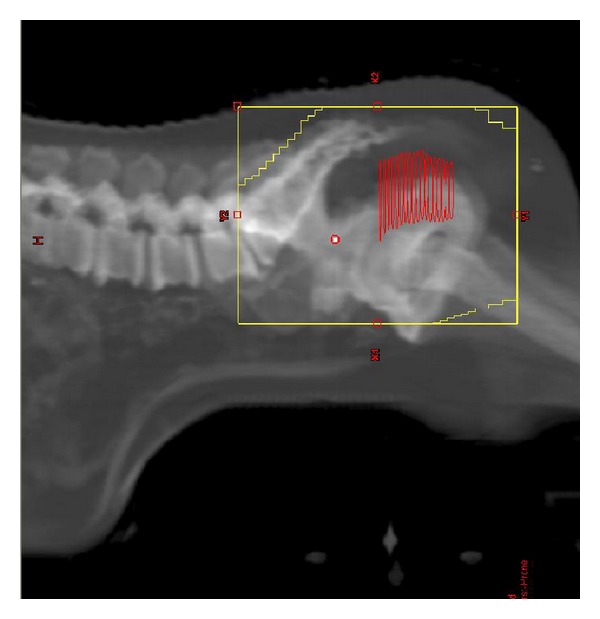
A bellyboard with a patient lying in a prone position is used to spare the intestine from RT. Red: gross tumor.

The patients were treated between June 2006 and March 2012. The criteria for analysis included patients with invasive rectal cancer, stage II, stage III, or low-lying stage I, who were treated with 3DRT that was designed to encompass the gross cancer in the rectum with margins and regional lymph nodes. All patients underwent CT scan simulation from L2 vertebral body to proximal femur at 3 mm slice thickness in a prone position on a bellyboard (Figure 1). Barium sulfate was infused in the rectum and radiopaque markers were placed at the anal verge prior to simulation. The CT simulation study was transferred to a treatment planning workstation (Varian Eclipse Treatment Planning System V10.0, Varian Medical Systems, Palo Alto, CA). The gross tumorous volume (GTV) in the rectum was identified and contoured. Three-field (posterior-anterior and laterals) or four-field (posterior-anterior, anterior-posterior, and laterals) RT techniques were used. For posterior-anterior and anterior-posterior fields, the superior border was at the L5/S1 junction, the lateral borders were at 1.5 to 2 cm lateral to the widest bony margin of the true pelvic side walls, and the inferior border was at least 3 to 5 cm inferior to the primary tumor or at the IBOF, whichever was more inferior. For lateral fields, the posterior border was at a minimum of 1.5 cm posterior to the posterior aspect of the sacrum and the anterior border was at the posterior margin of the symphysis pubis for T3 lesions and the anterior margin of the symphysis pubis for T4 disease. The superior and inferior borders were the same as defined in the posterior-anterior and anterior-posterior fields. Appropriate corner blocks were used to spare the muscle and soft tissues behind the sacrum and inferior to the symphysis pubis and intestine superiorly and anteriorly in the pelvis from unnecessary RT dose. The RT dose was calculated at the isocenter of the multiple fields and delivered at 180 cGy per fraction per day, five days a week, up to a total dose of 4,500 cGy in five weeks. This was followed with a tumor boost of 540 to 900 cGy at 180 cGy per fraction to cover GTV plus a 2-3 cm margin by opposed lateral fields or a three-field technique. A 10 MV photon beam was employed to deliver the RT. The EAS was retrospectively contoured in each case by a certified medical dosimetrist and later confirmed by a radiation oncologist and the volume was measured. The anatomic relationship between the EAS, IBOF, inferior RT field border, and inferior GTV edge was evaluated. The distances from the center of the EAS to the IBOF, the center of the EAS to the inferior GTV edge, and the center of the EAS to the inferior border of the RT field were measured. Dose-volume histograms were used to measure the RT dosimetric coverage of the EAS, including the mean, maximum, and minimum doses. The standard one-way analysis of variance (ANOVA) (*t*-test) was used to compare the significance of the differences.

## 3. Results

Characteristics of the patients are shown in Table 1. Of 64 consecutive patients, three had stage I, 11 had stage II, and 50 had stage III diseases. The inferior edge of the GTV ranged between 9 cm superior to and 1 cm inferior to the anal verge, with the average being 4.1 cm superiorly. Fifty-five (86%) patients received 5040 cGy and 9 (14%) received 5400 cGy.

The EAS was identifiable in all cases and was contoured in the planning CT scans. The mean EAS volume was 5.1 ± 1.9 cm^3^ (range, 1.3–11.2 cm^3^). On average, the center of the EAS was 19 ± 12 mm inferior to the IBOF. In 53 out of 64 cases (82.8%), the EAS was completely inferior to the IBOF. In the remaining 11 cases (17.2%), the EAS was either overlapping the IBOF (10 cases; 15.6%) or completely superior to the IBOF (1 case; 1.6%).

**Figure 2:**
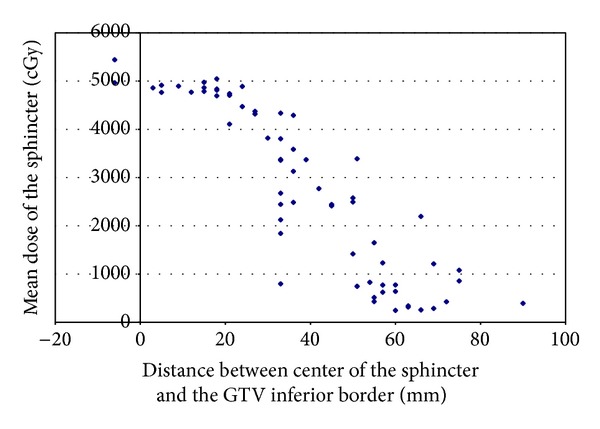
Mean dose of EAS versus distance between center of the EAS and the GTV inferior border.

**Figure 3:**
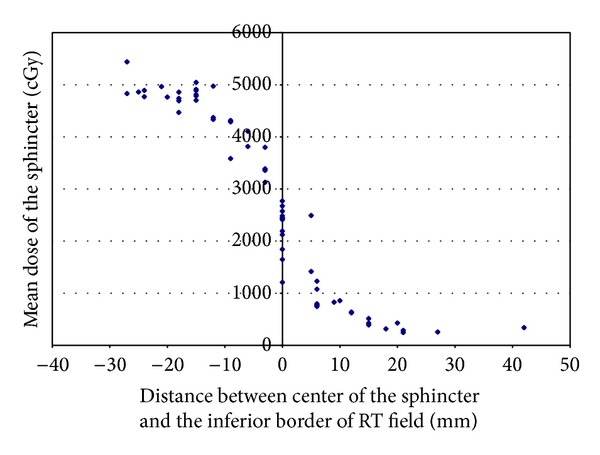
Mean dose of EAS versus distance between center of the EAS and the inferior border of the RT field.

**Figure 4:**
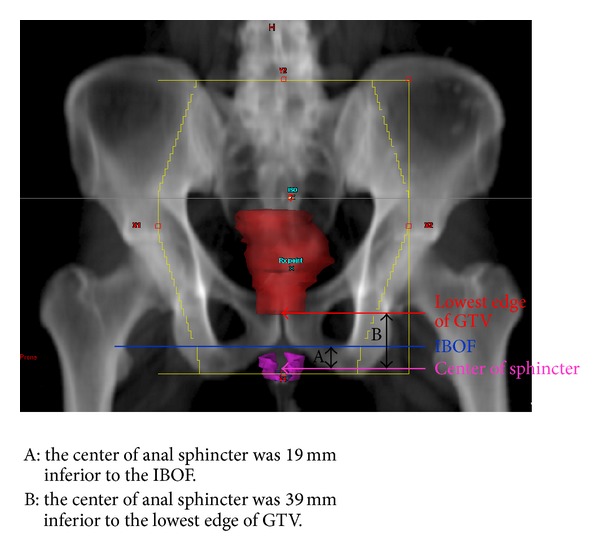
A typical case showing the anatomic relationship between the EAS, GTV, and pelvic bony landmarks for RT; solid red: GTV; magenta: EAS.

On average, the center of the EAS was 39 ± 22 mm inferior to the lowest edge of the GTV, ranging between 90 mm inferior to and 6 mm superior to the lowest edge of the GTV. On average, the center of the EAS was 2 ± 7 mm superior to the inferior border of the RT field, ranging between 42 mm inferior to and 27 mm superior to the inferior border of the RT field. In 19 cases (30%), the EAS was completely outside of the inferior border of the RT field. The average mean dose delivered to the EAS was 2795 cGy (range, 245–5441 cGy). Lower mean doses delivered to the EAS were noted for cases that had larger distances from the center of the EAS to the GTV inferior border (Figure 2). The average mean dose delivered to the EAS for cases with a distance more than 4 cm was 1264 ± 993 cGy, and for a distance less than 4 cm it was 4045 ± 1087 cGy (*P* < 0.00001). For cases where the center of the EAS was located more than 5 mm inferior to the field's inferior border, the mean EAS dose was 607 cGy. In contrast, for cases where the center of the EAS was located more than 5 mm superior to the field's inferior border, the mean EAS dose was more than 4000 cGy (*P* < 0.00001, Figure 3). A typical case showing the anatomic relationship between the EAS, GTV, and pelvic bony landmarks for RT is illustrated in Figure 4. Please note that in this case the EAS was completely distal to IBOF.

## 4. Discussion

To our knowledge, this is the first study to define the anatomic location of the EAS relative to pelvic bony landmarks by 3DRT in a prone position setup. This is also the first study to summarize the dosimetric coverage of EAS for patients with rectal cancer treated by 3DRT. Our data indicate that in all cases the EAS was identifiable in CT scans. In 82.8% of cases, the EAS was completely located inferior to the IBOF, which means, in majority of cases, that the EAS could be spared from unnecessary radiation exposure if the lower edges of the field were set at IBOF.

While the oncologic benefits of neoadjuvant pelvic RT for patients with rectal cancer have been confirmed by several randomized studies [1, 5, 6], there are only a few studies addressing the impact of RT on anorectal function. Certainly, cancer per se and surgery alone, such as total mesorectal resection, can compromise normal anorectal function and cause different levels of fecal frequency, urgency, incontinence, or emptying difficulties [7, 8]. It is generally believed that neoadjuvant RT could contribute a negative impact on anorectal function as well. Through a questionnaire survey by the Swedish Rectal Cancer Trial, compared to surgery-alone group, patients with preoperative short-course irradiation (5 Gy × 5) had a significantly worsened long-term bowel function including increasing bowel frequency, incontinence for loose stools, urgency, and emptying difficulties [9]. In addition, irradiated patients more commonly required a stoma later because of poor anorectal function after surgery. More importantly, 30% of irradiated patients reported an impaired social life that was related to bowel dysfunction, compared to 10% of the surgery-alone group.

In another questionnaire survey by Peeters et al. from the Dutch Colorectal Cancer Group Study [10], patients reported considerable long-term adverse effects of preoperative RT by 5 Gy × 5 on anorectal functional outcome. Compared with nonirradiated patients, irradiated patients had increased rates of fecal incontinence (62% versus 38%), had to wear a pad due to incontinence (56% versus 33%), and experienced anal blood loss (11% versus 3%) and mucus loss (27% versus 15%). In addition, satisfaction with bowel function was significantly lower in the irradiated group. While, from a radiobiological point of view, one can argue larger fraction size, 5 Gy per treatment, could be the reason for poorer chronic side effects, more recently, Bruheim et al. from Norway reported considerable long-term detrimental effects on anorectal function for patients receiving conventional long-course RT (2 Gy × 25 or 1.8 Gy × 28) [2]. In patients without a stoma, with a median time of 4.8 years since surgery, compared to patients who had surgery alone, a higher proportion of irradiated patients were incontinent for liquid stools (49% versus 15%), needed a sanitary pad (52% versus 13%), and lacked the ability to defer defecation (44% versus 16%). Poorer global quality of life and social function were also noted for these irradiated patients.

Indeed, irradiating normal structures could cause endovascular injury and overproduction of fibrogenic cytokines, such as transforming growth factors, which could lead to radiation-induced fibrosis as an end result [11]. Specifically, there has been quantitative evidence confirming sphincter dysfunction from pelvic RT for cervical, prostate, and anorectal cancers [12–16]. It is generally believed that anal canal pressure, especially basal resting pressure, could be reduced significantly by RT compared to baseline preirradiated state. In a prospective study for patients with mid and low rectal cancer, Ammann et al. compared anorectal manometric values before and after surgery [16]. Anorectal manometry was performed preoperatively and at a median of 383 days postoperatively. The mean resting pressure for patients with neoadjuvant CRT decreased significantly from 89 ± 35 mm Hg preoperatively down to 53 ± 17 mm Hg postoperatively. In contrast, no statistically significant manometric differences occurred before and after surgery for patients who underwent surgery alone. Therefore, shielding of the anal sphincter was recommended whenever a sphincter-preserving procedure was considered.

Radiation portals for patients with rectal cancer are generally defined by anatomic locations of cancer, lymphatic pathways of drainage, and patterns of locoregional cancer recurrence. Reviewing patterns of failure in 75 rectal cancer patients using second or symptomatic look operations following curative surgery, Gunderson and Sosin found that local failure and/or regional lymph node metastases occurred as the only failure in nearly 50% of the failure group [17]. The benefits of radiation treatment were suggested, and, based on failure patterns, appropriate radiation portals were defined. Specifically, it was suggested that the inferior border of the field be set at 3 to 5 cm inferior to the primary tumor or at the IBOF, whichever was more inferior [4, 17]. The current standard is to use a multiple beam setup and the borders of the fields are determined based on the anatomic locations of cancer and pelvic bony landmarks.

**Figure 5:**
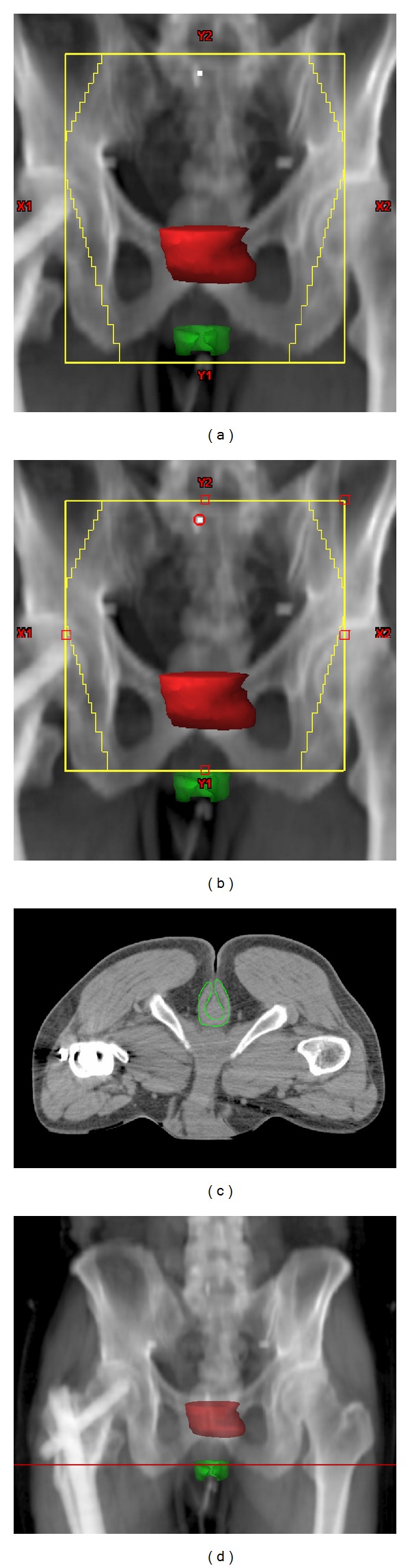
A patient with a rectal cancer located 2.7 cm above the EAS. (a) The original RT field covered 4 cm inferior to the gross tumor. (b) The EAS can be spared from radiation treatment easily by reducing the inferior border superiorly by 2 cm. (c) Axial CT image through the EAS shows that no draining lymph nodes need to be covered. (d) Coronal scout view shows the level of axial image (red line). Solid red: gross tumor; green: EAS.

With the increasing need to implement target-directed RT for better results, recently, consensus on structures that should be included in the target volume for patients with rectal cancer has been defined [18, 19]. Based on the recurrence data from a systematic review of 18 studies, Roels et al. concluded that the primary tumor, the mesorectum, and the presacral and internal iliac nodal areas should be covered in all cases [18]. With respect to the inferior extent of the target volume it is agreed that a minimum of 2 cm inferior to the gross disease is needed and the entire mesorectum to the pelvic floor should be included even for cases with upper rectal cancer [19]. Mesorectum is the mesentery with lymphovascular and neural structures supporting and connecting the midupper portion of the rectum with the sacrum. It is cylindrical in shape starting at the level of sacral promontory and ending at the level where the levator ani muscle meets with the rectal wall. For patients with positive pelvic lymph nodes, by systematic review, the mesorectal lymph nodes were involved in up to 87% of the cases [18]. After evaluating surgical specimens in 311 consecutive patients with colorectal cancer, Morikawa et al. reported that no lymphatic metastasis was noted more than 4 cm distal to the tumor in the mesorectum [20]. To define the inferior border of RT fields, in addition to the anatomic location of the primary tumor and the extent of mesorectum, the internal iliac lymph nodes, specifically the areas with obturator lymph nodes, need to be taken into consideration. Inferiorly, it is recommended to include the obturator lymph nodes till the level where the obturator artery enters the obturator canal [18]. In any case, because the EAS is located inferior to the levator ani and in the current study in 98.4% of cases the EAS was either completely inferior to (82.8%) or overlapping with (15.6%) the IBOF, theoretically, fields of 3DRT should be designed to spare the EAS for mid/upper rectal cancer where there is no evidence of EAS cancerous involvement. Of note, for cases in this study that had the tumor located more than 4 cm above the EAS, the EAS was spared from unnecessary radiation (average mean dose 1264 cGy) in the majority of cases, even though the EAS was not identified in the original RT planning. If the EAS was identified and RT plans were designed to avoid it, it is likely that the dose of RT to the EAS would be even lower in such a patient population. For cases where the tumor is located within 4 cm of the EAS, given that margins are needed to add to clinical target volume to account for setup variation, it will not be easy to spare EAS by conventional 3DRT. However, with daily image guidance to reduce the need of adding planning target margins and by designating the EAS as an avoidance structure, sparing the EAS from RT would be possible in certain cases that have the cancer located 2 to 4 cm superior to EAS (Figure 5). Apparently, if the cancer is within 2 cm of the EAS, or an abdominoperineal resection is planned after neoadjuvant CRT, the EAS should be considered as part of RT targets and covered.

One of the limitations of the current study is that we were not able to define the internal anal sphincter by the CT scans. It is essential to point out that while sparing the EAS from unnecessary RT is recommended when it is feasible, the cephalad extent of the internal anal sphincter is at the level of the caudad extent of the mesorectum and, therefore, at least the upper portions of the internal anal sphincter will inevitably be included in the RT fields. Another limitation is that the correlation between RT dose distribution to the EAS and anal-rectal dysfunction was not studied, which will be the focus of our future studies.

## 5. Conclusions

In summary, we have evaluated the anatomic location of the EAS to pelvic bony landmarks related to 3DRT and studied the dosimetric coverage of the EAS in patients undergoing neoadjuvant CRT for rectal cancer. 3D planning under CT guidance allows accurate delineation of the EAS. In 82.8% of patients, the EAS is located completely inferior to the IBOF. Because RT can cause anorectal dysfunction, meticulous planning to define the inferior border of the RT field is recommended to spare the EAS.

## Figures and Tables

**Table 1:** Characteristics of the patients.

Characteristics	Number
Number of patients	64
Staging	
T2N0	3
T2N1	2
T3N0	9
T3N1	45
T3N2	2
T4N0	2
T4N1	1
GTV to anal verge	
Mean	4.1 cm
Range	−1 to 9 cm
RT dose	
5040 cGy	55
5400 cGy	9

## Abbreviations

EAS: External anal sphincter
3DRT: 3-Dimensional conformal radiotherapy
GTV: Gross tumorous volume
IBOF: Inferior border of the obturator foramen
CRT: Chemoradiation
RT: Radiotherapy.
